# Comparative Morphology of the Symbiont Cultivation Glands in the Antennae of Female Digger Wasps of the Genus *Philanthus* (Hymenoptera: Crabronidae)

**DOI:** 10.3389/fphys.2022.815494

**Published:** 2022-01-26

**Authors:** Wolfgang Goettler, Martin Kaltenpoth, Samuel McDonald, Erhard Strohm

**Affiliations:** ^1^Department of Zoology, University of Regensburg, Regensburg, Germany; ^2^Department of Insect Symbiosis, Max Planck Institute for Chemical Ecology, Jena, Germany; ^3^Swiss Light Source, Paul Scherrer Institute, Villigen, Switzerland

**Keywords:** 3D-reconstruction, micro-CT, morphology, symbiosis, *Streptomyces*, actinomycetes, *Philanthus*

## Abstract

Females of the solitary digger wasp tribe *Philanthini*, called the beewolves (Hymenoptera, Crabronidae), cultivate strains of symbiotic bacteria that belong to the genus *Streptomyces* in unique and highly specialized glands in their antennae. The glands consist of large reservoirs that are surrounded by numerous gland cell complexes (class III). The symbionts are cultivated inside the reservoirs and are probably provisioned with nutrients secreted from the surrounding glands and/or sequestered from the hemolymph. The wasp female delivers the bacteria into the subterranean brood cell prior to oviposition. Fully grown larvae take up the bacteria and apply them to their cocoon. There the bacteria produce several antibiotics that protect the wasp offspring against fungus infestation. Hitherto *Streptomyces* bacteria were detected in the antennae of 38 species of the Philanthini. However, a detailed morphological analysis of the antennal glands is only available for a few species. In order to shed light on the evolutionary history of the association between beewolf wasps and bacteria, we investigated the morphology of the antennal glands of another 14 *Philanthus* species from the Palearctic, Paleotropic, and Nearctic. We generated 3D-models of the glands based on serial semithin sections and/or micro-CT (μCT). Despite broad similarities in number and structure of antennal glands, the results revealed interspecific differences with regard to overall shape, complexity, and relative size of the reservoirs as well as the number of the surrounding gland cell units. Mapping the morphology of all species studied so far on the phylogeny (that parallels geographical distribution) revealed that related species share similarities in gland morphology, but there are notable differences between lineages. In particular, compared to the North American species the European and African species possess more complex gland structures with a higher number of gland cells. We discuss morphological, ecological, and physiological aspects and provide scenarios for the evolution of the antennal glands of the *Philanthini* as symbiont cultivation organs.

## Introduction

Symbioses with microorganisms are crucial for development, survival and reproduction of a large number of organisms (McFall-Ngai et al., 2013). In insects, symbionts are often cultivated in specialized cells or organs and provide essential nutrients or protection against pathogens or predators (Douglas, 2015). A unique protective symbiosis has been reported between several species of beewolf wasps (Hymenoptera, Crabronidae, Philanthinae, and Philanthini) and bacteria of the genus *Streptomyces* (Actinomycetales, Streptomycetaceae). Beewolf females cultivate the bacteria in large gland reservoirs in five segments of each of their antennae (Strohm and Linsenmair, 1994/95; Kaltenpoth et al., 2005, 2014a; Goettler et al., 2007). Inside the reservoirs, the bacteria are likely provided with nutrients by secretions of associated gland cells as well as substances that are sequestered into the gland lumen from the hemolymph (Kaltenpoth et al., 2005, 2006; Goettler et al., 2007; Nechitaylo et al., 2014, 2021). Prior to oviposition, female beewolves secrete readily visible amounts of the pasty, whitish mixture of bacteria and lipids (Kaltenpoth et al., 2009) to the ceiling of the subterranean brood cells (Strohm and Linsenmair, 1994/95).

For the European beewolf, *Philanthus triangulum*, it has been shown that this “white substance” serves two functions. First, since brood cells are often located deep in the soil (in *P. triangulum* up to 1 m from the surface) beewolf offspring need directional information for successful and secure emergence (Strohm and Linsenmair, 1994/95). The female deposits the antennal secretion at the distal side of the brood cell. Larvae locate the secretion and attach their cocoon in such a way that their head points to the main burrow that remains open after the completion of the brood cell. After eclosion from the cocoon, they move forward in this direction, find the main burrow, and emerge from the soil. Thus the secretion provides an orientational cue for the larvae (Strohm and Linsenmair, 1994/95). Second, the secretion is taken up by the larvae at the beginning of cocoon spinning and the bacteria are embedded into the silk threads of the cocoon. There the bacteria produce nine different antibiotics that protect the cocoon from infestation by mold fungi that would otherwise infest the cocoon and kill the larvae inside (Kaltenpoth et al., 2005, 2006; Kroiss et al., 2010; Engl et al., 2018). Notably, altogether nearly 50 different antibiotics were found in a study on 25 *Philanthus* species, strongly supporting the defensive role of the symbionts (Engl et al., 2018).

The symbiotic bacteria associated with the wasps of the tribe Philanthini constitute a monophyletic group within the genus *Streptomyces* (Kaltenpoth et al., 2006) and have coevolved with their hosts (Kaltenpoth et al., 2014a). However, details of the morphology of the antennal glands that provide the structural basis for the symbiosis between beewolves and *Streptomyces* bacteria have only been provided for a few species (Goettler et al., 2007; Kaltenpoth et al., 2010, 2012). How the complex glands might differ among species and how they might have evolved has not yet been investigated. Here, we provide a comparative analysis of the morphology of the females’ antennal glands of 15 *Philanthus* species (14 for the first time, plus *P. triangulum* for direct comparison) in order to shed light on the variability, evolutionary origin, and subsequent changes in these glands.

Members of the Philanthini (*ca*. 172 species) are distributed worldwide with the exception of Australia and Antarctica (Bohart and Menke, 1976; Evans and O’neill, 1988). The tribe comprises the genera *Philanthus* (*ca*. 137 species), the South American genus *Trachypus* (*ca*. 31 species, might actually group within and thus belong to the genus *Philanthus*), and the genus *Philanthinus* (four species, Pulawski, 2018). Bacteria of the genus *Streptomyces* have been found in the antennae of all of the 39 members of the Philanthini investigated so far (comprising 34 *Philanthus*, four *Trachypus*, and one *Philanthinus* species) covering the whole distribution including the Palaearctic, Palaeotropics, Nearctic, and Neotropics. However, no such bacteria were found in the antennae of species of the related tribes Cercerini and Aphilanthopsini (Kaltenpoth et al., 2005, 2012; for the phylogeny of the subfamily see Alexander, 1992). This suggests that the defensive symbiosis occurs throughout the Philanthini, but is restricted to this tribe. How widespread the other function mentioned above, the orientational cue for the larvae (Strohm and Linsenmair, 1994/95), is among all these species is not known. In any case, considering the costs of the structure and of cultivating the bacteria, the symbiosis with the *Streptomyces* bacteria appears to be an important adaptation for the Philanthini.

**Figure 1:**
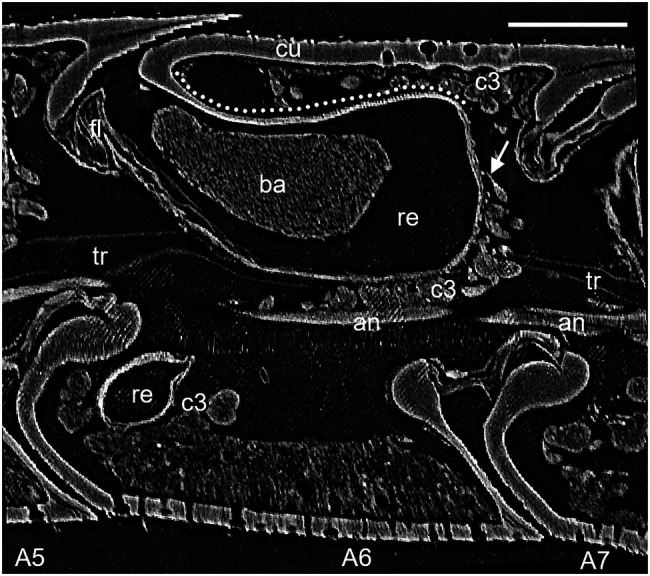
Lateral view of micro-CT (μCT) data of antennomere A6 of a female *Philanthus ventilabris*; an, antennal nerve; ba, bacteria; c3, class 3 gland cells; cu, outer cuticle; fl, flap mechanism; re, reservoir; tr, tracheole; arrow – conducting canal; and dotted line – reinforced cuticle. Scale bar 100 μm.

The antennal glands of the European beewolf *P. triangulum*, the South American *Trachypus boharti* and *Trachypus denticollis*, and *Philanthinus quattuordecimpunctatus* from Turkey have been previously described in detail (Goettler et al., 2007; Kaltenpoth et al., 2010, 2012) and show largely similar morphologies. In *P. triangulum* and the two *Trachypus* species, the symbiont cultivation organs occur in five antennal segments (=antennomeres), whereas *P. quattuordecimpunctatus* houses the symbionts in six antennomeres. The gland reservoirs have complex shapes with a main part and a more or less distinct lobe connected by a restriction (Goettler et al., 2007). The reservoirs are formed by invaginations of the cuticle at the proximal, dorsal side of the antennomere. The cuticular walls of the glands are surrounded by an epithelium bearing class III (Noirot and Quennedey, 1974) gland cell units each of which consists of a secretory cell that is connected to the lumen by a canal cell. Several of these gland cell units are grouped in so-called acini. The opening on the dorsal proximal side is closed by an elastic plug (see Goettler et al., 2007; Figure 1).

We hypothesized that by comparative morphological analysis of several *Philanthus* species some intermediate states might be revealed. Mapping these onto a phylogenetic tree would help to elucidate the evolution of these unique symbiont cultivation organs. Moreover, the ecological relevance of the glands with regard to the defense of the cocoon against microbes might vary across species. This could be reflected by the relative size of the gland. Thus, we investigated the morphology of the antennal glands of females of 15 *Philanthus* species. We created 3D-models of the glands based either on serial histological semithin sections or on datasets obtained by micro-CT (μCT). We compared overall gland structure, estimated gland size, and numbers of associated gland cells. We discuss the morphology of the gland and the associated gland cells with regard to ecological, physiological, and evolutionary aspects.

## Materials and Methods

### Specimens

The antennae of females of 15 *Philanthus* species comprising two European, two African, and 11 North American species were investigated. All specimens were caught in the field, cold anaesthetized and fixed (see below). For seven *Philanthus* species, 3D reconstructions of one specimen were generated based on digital images obtained by light microscopy of serial sections (number of histological specimens available given in brackets): *Philanthus melanderi* (1), *Philanthus rugosus* (1) (Western and Eastern Cape Province, South Africa, respectively), *Philanthus coronatus* (1) (Freiburg, Germany), *Philanthus basilaris* (2), *Philanthus gloriosus* (3), *Philanthus pacificus* (1) (San Rafael Desert, Utah, United States), and *Philanthus bicinctus* (4) (Wyoming, United States). For another eight *Philanthus* species, we additionally reconstructed the antennal glands based on microtomography (μCT, one specimen each) data (number of histological specimens available given in brackets): *Philanthus albopilosus* (1), *Philanthus barbiger* (1), *Philanthus crotoniphilus* (1), *Philanthus parkeri* (1), *Philanthus psyche* (1) (San Rafael Desert, Utah, United States), *Philanthus multimaculatus* (1), *Philanthus ventilabris* (1) (Salt Lake City, Utah, United States), and *P. triangulum* (12) (Würzburg, Germany).

### Semithin Sections

Preparation of female antennae was performed as described in Goettler et al. (2007). In brief, antennae were fixed with either Carnoy’s solution (ethanol/chloroform/glacial acetic acid, 6:3:1) or alcoholic Bouin (Romeis, 1989), dehydrated in a graded ethanol series (10 min in 70, 80, 90, 96, and 100%, respectively) embedded in Poly/Bed® 812 according to the manufacturer’s instructions (Polysciences, Eppelheim, Germany). Serial sections of 4 μm thickness were cut with a diamond knife on a Reichert 2040 Autocut microtome and stained with 1% toluidine blue buffered with 1% Di-sodium-tetraborate in distilled water. Digital images of the serial sections of the antennae were obtained with a Nikon DS-2Mv camera attached to a Zeiss Axiophot microscope at the appropriate magnification.

### Micro-Computer-Tomography

For X-ray μCT, female antennae were fixed in a solution of 4% glutardialdehyde in 0.1 M sodium-cacodylate-buffer, pH 7.2. After dehydration in a graded ethanol series (see above) and transfer to acetone, the specimens were critical point dried (BAL-TEC CPD 030) and kept in an exsiccator until μCT scanning. The μCT scans were performed at the Swiss Light Source (SLS), which is part of the Paul Scherrer Institute (PSI) in Villigen, Switzerland. In each of the dried antennae, antennomere A6 was scanned with high-energy X-rays at the X02DA-TOMCAT beamline of the electron accelerator. Each antenna was mounted upright at its proximal segment on a metal needle and positioned orthogonally to the X-ray beam. During the 6 min scan, the antenna was rotated by 180° around its longitudinal axis. Post scanning processing resulted in a cubic 3D-data set of 757 μm edge length with a resolution of 1,024 volumetric pixels (=voxels). All voxels were isotropic with an edge length of 0.74 μm in each spatial direction.

### 3D-Reconstruction

Both, serial semithin sections and μCT data sets were used to reconstruct the morphology of the glands using the visualization-software Amira® (Mercury Computer Systems, Berlin). The images of the semithin sections were stacked and aligned (automatic alignment with manual correction). This step was not necessary for the μCT data. In each semithin section and μCT layer of antennal segment 6, the borders of the gland reservoirs, flap, and antennal nerves and for specimens investigated by μCT and also the gland cells were marked manually with different colors. Then the software calculated the 3D-model of these structures. Whereas in the μCT data sets the voxels, where isotropic (1 × 1 × 1) voxel-size in the semithin sections had a ratio of about 1 × 1 × 10 due to lower resolution in z-axis (thickness of the section 4 μm) compared to the xy-plane. To reveal possible differences between the 3D-reconstruction of semithin sections and μCT data sets, we compared the 3D-model based on μCT data of *P. triangulum* with a reconstruction by means of semithin sections that had been published earlier (Goettler et al., 2007).

### Measurements

The volume of a gland reservoir is strongly affected by its filling status. When filled with secretion and bacteria, antennal reservoirs are bulged out on one side, whereas they are much thinner after the female digger wasp secreted the content into the brood cell (Goettler et al., 2007). The area of the reservoir surface, by comparison, is much less affected by filling status so we considered this as a measure of gland size. Moreover, since species differ considerably in body size, we calculated surface area of the antennal gland reservoirs relative to the surface of the respective antennomere. The reservoir surface area was computed with Amira® software. The outer surface of the antennomere was estimated by assuming that it is a cylinder and after measuring its length (*l*_ant_) and radius (*r*_ant_), we calculated the surface area according to the formula *A*_ant_ = *r*_ant_^2^ π *l*_ant_.

## Results

### Overall Morphology

**Figure 2:**
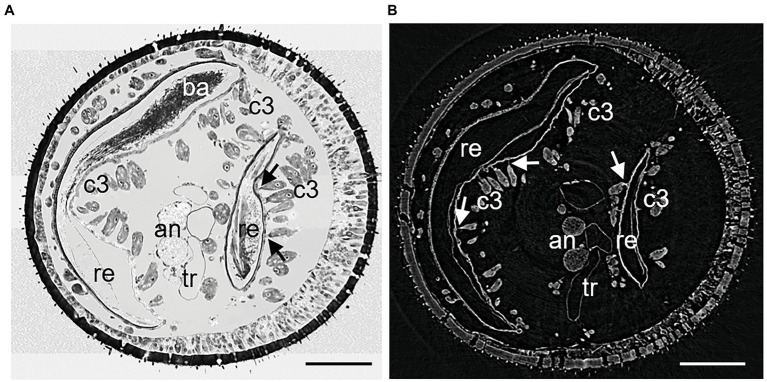
Cross sections of similar areas of antennomeres A6 of female *Philanthus triangulum*: **(A)** Semithin section and **(B)** μCT image. The larger part of the reservoir is on the left, the smaller lobe on the right. The cuticle on the left of each reservoir part is thickened and reticulated. Canals of the class III gland cell units are readily visible also in the μCT image. Due to critical-point drying, cells and tissues in **(B)** may have shrunk. an, antennal nerve; ba, bacteria; c3, class 3 gland cells; re, reservoir; tr, tracheole; and arrows – conducting canals between acini and reservoir. Scale bars 100 μm.

The denotation of antennal segments follows the suggestion of Isidoro et al. (1996) with the scapus representing A1, pedicellus A2, and A3–A12 the flagella. All *Philanthus* species that were investigated in this study had antennal glands in the five antennomeres A4–A8. We did not notice any conspicuous differences in the overall morphology of the five single glands within one antenna in any species. Therefore, for all species, we used segment A6 for detailed reconstruction (Figures 1, 2). The resolution and contrast of the μCT data set was sufficient to reveal morphological details and even single cells (Figures 1, 2). We did not find any striking differences between histology and μCT investigation with regard to location and shape of glands and other tissues within the antennae. However, in the μCT data set, soft tissues and cells showed a reduced volume, which is probably an artifact caused by the critical-point drying process (Figures 1, 2).

In all species under study, the antennal gland reservoirs represented an invagination of the cuticle at the proximal and dorsal side of the antennomere (Figures 1, 2). On the outer dorsal and the outer lateral side, the cuticle of the reservoir was thickened and it showed a net-like structure (Figures 1, 2). *In vivo* the dorsal opening of the reservoir was covered by the adjacent proximal segment and was only visible when the antenna was bent downward. A fibrous structure reached into the opening of the reservoir from the inner side. This probably acts as an elastic plug mechanism that closes the reservoir when no secretion is delivered but is pushed aside due to an increased hemolymph pressure when the white substance is being secreted (Figure 1; see also Goettler et al., 2007). In all reservoirs of the different specimens, filamentous bacteria could be detected, but specimens differed in the filling status of the glands.

### Gland Reservoirs

The 3D-reconstruction revealed some interspecific variation with regard to the shape and the relative size of the antennal gland reservoirs. All reservoirs had an opening at the proximal dorsal side of the antennomere and extended apically and laterally as more or less bent lobes. The length of the gland ranged between ½ and ¾ of the length of the antennomere. There was no significant correlation between the surface area of the reservoir and the length of the antennomere (Supplementary Figure 1) or of the antenna (Supplementary Figure 2).

**Figure 3:**
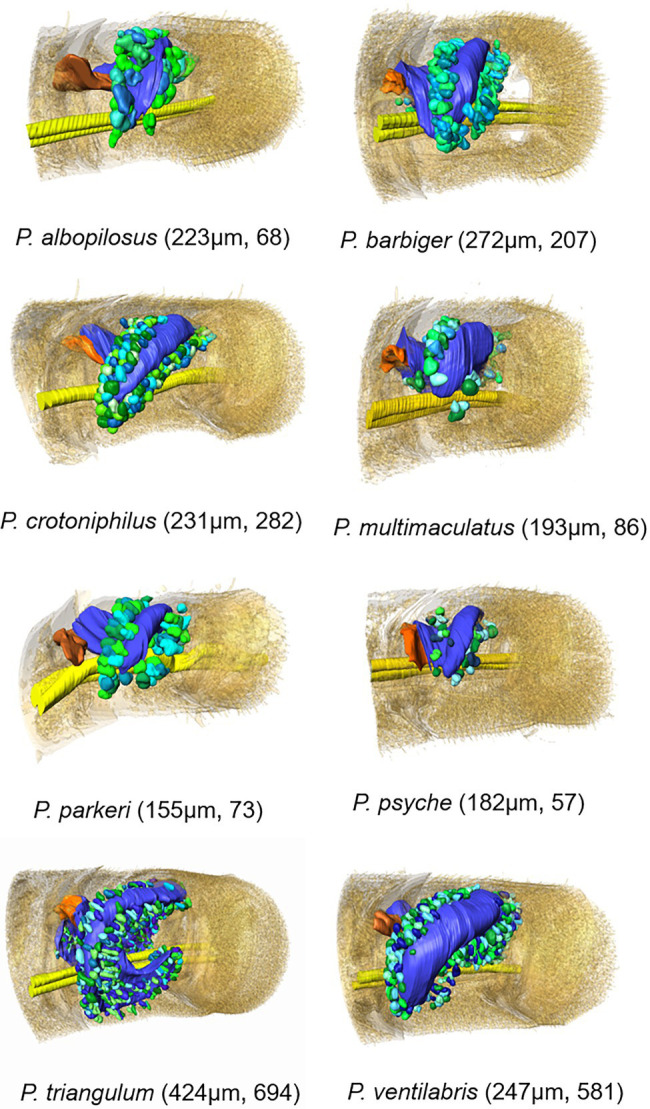
3D-models based on μCT data sets of antennal glands in antennomere A6 of females of eight *Philanthus* species with surrounding gland cell units (length of the respective antennomere and total number of acini in brackets). Color code: different green and turquoise hues: acini; yellow: antennal nerve; orange: flap; and blue: gland reservoir; the outer antennomere cuticle is displayed half transparent.

The 3D models revealed that all African and European species (*P. coronatus*, *P. melanderi*, *P. rugosus*, and *P. triangulum*) had complex reservoir structures with two flattened sections located more or less cylindrically around the axis of the antennomeres (Figure 3; Supplementary Figure 3). The main part, with the opening, was located at the dorsal and lateral side of the antennomere and had a more or less constricted connection to a lobe that extended to the medial side of the antennomere. *Philanthus triangulum* showed the most complex reservoir structure of all species investigated, so far since the medial lobe was u-shaped and comparatively large (Figure 3; Supplementary Figure 3; see also Goettler et al., 2007). The North American species (*P. albopilosus*, *P. barbiger*, *P. basilaris*, *P. bicinctus*, *P. crotoniphilus*, *P. gloriosus*, *P. multimaculatus*, *P. pacificus*, *P. parkeri*, *P. psyche*, and *P. ventilabris*) in contrast showed smaller, more sac-like gland reservoirs with less prominent or even lacking medial lobes (Figure 3; Supplementary Figure 3).

The 15 *Philanthus* species showed a median relative reservoir surface area of 46% (1st quartile: 42, 3rd quartile: 52.5, range: 19.5–38.7%) of the respective antennomeres’ surface areas. One North American (*P. ventilabris* 68%) and two European/African species (*P. coronatus* 67%, *P. triangulum* 71.2%) had comparatively high relative reservoir surface areas (Figure 3; Supplementary Figures 1–3).

### Gland Cells

In all species under study, the antennal gland reservoirs were surrounded by gland cells of the class III type (Noirot and Quennedey, 1974), which were clustered in spherical or club-shaped acini. The acini consisted of 2–9 single class III gland cell units with connections to the reservoir by conducting canal cells. The 3D models of all species investigated with μCT showed that the acini were arranged more or less densely in a belt around the reservoir (Figure 3). Only in *P. triangulum* the acini were more evenly distributed over the reservoirs’ surface. The total number of the acini could be reliably determined in the μCT data sets and varied between 57 (*P. psyche*) and 694 (*P. triangulum*; Figure 3). The number of acini significantly positively correlated with the surface area of the reservoir (Supplementary Figure 4) as well as with the length of the antennomere (Supplementary Figure 5).

### Phlyogenetic Trends

**Figure 4:**
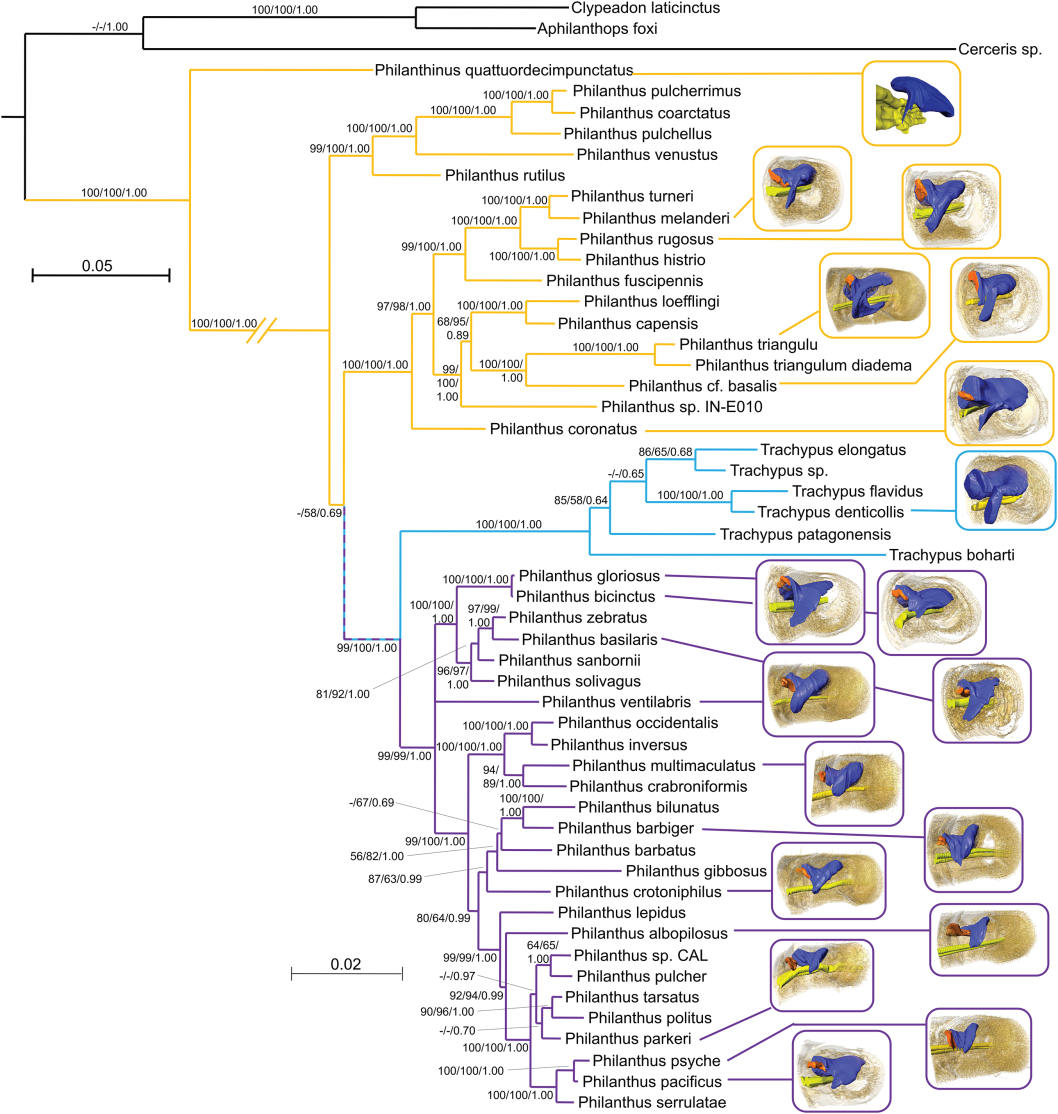
Mapping of the available 3D reconstructions onto a phylogenetic tree of the Philanthinae. Yellow lines link European/African/Asian species, Light blue lines link South American species, and Lilac lines link North American species. Node values represent bootstraps from PAUP*, RAxML, and posterior probabilities from MrBayes, respectively (for details, see Kaltenpoth et al., 2014a,b).

Phylogenetic relationships (Kaltenpoth et al., 2014b) are paralleled by the geographical distribution of the species. Thus, the differences among species from different regions as stated above largely apply to the phylogenetic groups (Figure 4). Antennal glands of females of the African and European clade of *Philanthus* (*P. triangulum*, *Philanthus coconatus*, *P. rugosus*, *P. melanderi*, and *P. coronatus*) had more complex overall shapes in that they possessed a more or less pronounced second lobe. Whereas most North American species had only one lobe (*P. multimaculatus*, *P. crotoniphilus*, *P. albopilosus*, *P. parkeri*, *P. barbiger*, *P. psyche*, and *P. pacificus*), a second group of North American species that belong to a distinct clade (*P. ventilabris*, *P. gloriosus*, *P. basilaris*, and *P. bicinctus*) showed at least some extension of the main lobe. The glands of the European *P. quattuordecimpunctatus* resembled the North American species, while the South American *Trachypus* species showed more similarities to the European and African lineages.

## Discussion

### Morphology

Classical histological as well as modern μCT methods revealed that the females of all 15 *Philanthus* species under study (14 studied for the first time, plus *P. triangulum*) possess antennal glands that function as symbiont cultivation organs in five of their antennomeres (A4–A8).

The use of μCT scans in this study turned out to be a suitable method in particular to create 3D reconstructions. Specimen preparation for μCT is comparatively simple and the non-destructive scans revealed anatomical details without the risk of lost or deformed slices or inadequate staining as it might occur for classical histological methods. The lower x–y resolution of the μCT-scans compared to light microscopy of semithin sections is sufficiently compensated by a high z-axis resolution and the fact that the tomographic data sets need no alignment. However, we recognized some tissue shrinkage in the critical-point dried specimens, which is probably due to sample preparation and not due to the μCT scanning. This artifact was, however, not relevant for the objective of this study.

The basic structure of the antennal glands was similar across all Philanthini investigated in this and in earlier studies (Goettler et al., 2007; Kaltenpoth et al., 2010, 2012). There was an invagination of the proximal dorsal cuticle of the antennomere surrounded by an epithelium. The structure of the cuticular walls of the reservoir appeared to be very similar as well in having a thin inner cuticle and a thicker and reticulated wall toward the lateral side. The net like structure of the wall probably facilitates sequestration of nutrients from the wasp female’s hemolymph into the gland reservoir. Moreover, all species studied so far showed complex class III gland cells (Noirot and Quennedey, 1974) grouped in acini associated with the reservoir. These glands cells might provide the bacteria with essential nutrients like chitobiose, certain amino acids, and vitamins (Nechitaylo et al., 2014).

Despite their overall similarity, there are gradual differences in the morphology of the glands across species that are largely related to their geographic origin and, thus, their phylogenetic position (Figure 4). The four investigated *Philanthus* species from Africa and Europe showed a more complex structure of their gland reservoirs compared to the 11 North American species. This corroborates the results from the phylogenetic analysis that new-world and old-world species represent distinct subgroups within the genus (Kaltenpoth et al., 2014a). However, the glands of the South American *Trachypus* species (Kaltenpoth et al., 2012) appear to resemble the European/African species rather than the more closely related North American species in having a pronounced second lobe of the reservoir. The European *Philanthinus* (Kaltenpoth et al., 2010), in turn, stands out for having glands in six rather than five antennomeres but otherwise resembles the North American species by showing no second lobe. It appears that the addition of a gland in another antennomere and the gain or loss of the second lobe of the reservoir are evolutionary somewhat flexible modifications of the basic organization of the antennal glands.

### Ecological and Physiological Aspects

Notably, there is a conspicuous pattern across species with regard to gland complexity and physiology of their respective symbionts. Compared to the species with a less sophisticated gland morphology (*Philanthinus* and the monophyletic North American *Philanthus*), species that possess relatively large and complex glands (Eurasian/African *Philanthus* and the South American *Trachypus*) harbor bacterial strains that need more nutrients and are more susceptible to antibiotics (Nechitaylo et al., 2014). It might be hypothesized that this is due to a higher degree of interdependence between these wasp species and the respective bacterial strains. Possibly, species with a relatively high rate of brood cell production and with a relatively high risk of infestation of their cocoons might require more bacteria, thus generating selection for larger glands and closer association with their symbionts. However, it might be questionable to draw conclusions about the amount of *Streptomyces* bacteria that are cultivated and secreted based on morphological characters like relative reservoir volume or number of gland cells. We have estimated the growth rate of the bacteria in *P. triangulum* (Kaltenpoth et al., 2010) but it is not clear whether these results can be transferred to other species. Thus, how the differences in the size of the reservoirs and the abundance relate to the possibly differing requirement of the cocoons for protection cannot be answered yet.

### Evolutionary Aspects

The *Philanthus-Streptomyces* symbiosis was estimated to be about 68 million years (my) old (Kaltenpoth et al., 2014a,b). This is relatively young compared to the age of the aphid-*Buchnera* symbiosis with 160–280 my (Moran et al., 1993) or the mutualisms of cockroaches and termites with their symbionts of the *Flavobacterium-Bacteroides* group with 135–250 my (Bandi et al., 1995).

All 39 species of Philanthini investigated so far with genetic methods bear bacteria in their antennae and the symbionts form a monophyletic group within the genus *Streptomyces* (Kaltenpoth et al., 2014a,b). However, the other tribes within the subfamily Philanthinae that have been studied lack the conspicuous antennal glands and symbiotic *Streptomyces* bacteria. This suggests that the symbiosis and the antennal glands evolved in the ancestor of and are restricted to the tribe Philanthini, thus representing an autapomorphy. Most likely the bacteria are used throughout the tribe in a similar way to protect the offspring from fungal infestation. This is corroborated by the occurrence of antibiotics on the cocoons of all of the 25 *Philanthus* species studied in this regard (Engl et al., 2018).

How widespread the use of the antennal gland secretion as orientational cue for the progeny among the *Philanthini* is beyond *P. triangulum* (Strohm and Linsenmair, 1994/95), is not yet known. Although some species are confronted with similar problems as *P. triangulum* of finding their way out of large subterranean nests, there are species that have shallow nests in a sandy soil that is not too hard (Evans and O’neill, 1988). This would allow the emerging offspring to simply move upward from their brood cells. Thus, some species might have lost the orientational function of the secretion.

Generally, antennal glands let alone complex ones as described here for female Philanthini seem uncommon among Hymenoptera. Some ant workers and queens (Isidoro et al., 2000; Renthal et al., 2008) as well as male parasitoid wasps (see e.g., Romani et al., 2008 and references therein) have pores on some antennal segments, sometimes with enlarged antennomeres and associated gland tissue (Heinze et al., 2021). For males a role in courtship is assumed (Bin et al., 1999; Romani et al., 2003; Heinze et al., 2021). For females the function is unknown. A role as propaganda chemical in a slave making ant or as source of antimicrobials has been proposed (Romani et al., 2006).

However, a diverse range of organs for the extracellular cultivation of protective symbionts in other parts of host insects have been described. In attine ants, different levels of complexity of structures for the cultivation of symbiotic bacteria have been reported (Currie et al., 2006; Li et al., 2018). In some species, bacteria occur on the legs and antennae, with no specialized structures. Other species evolved dedicated crypts or tubercles that are associated with glands (Currie et al., 2006; Li et al., 2018). In species of the beetle subfamily Lagriinae, bacteria are cultivated in adults in accessory glands associated with the reproductive tract, whereas in their larvae the symbionts are kept in invaginations of the dorsal cuticle (Flórez et al., 2017). In attelabid weevils, antibiotics producing fungi are cultivated in ventral mycangia (Kobayashi et al., 2008). In the latter two cases gland cells that provide nutrients for the symbionts might be involved as well.

As in the above-mentioned cases, the complexity of the antennal organs for the cultivation of symbionts as well as the symbiosis itself call for a scenario for their evolution in the Philanthini (Goettler et al., 2007). Different hypotheses are thinkable. (1) One plausible scenario might start with a simple antennal gland as the initial stage. Considering homology criteria, such an ancestral gland should have consisted of class III gland cells that were located at the dorsal, proximal part of the antennomere. Actually, we have evidence for the occurrence of such gland units in the antennae of both sexes of Philanthinae wasps (Strohm, in prep.). The function of these tiny glands is not known. Their location suggests that they secrete substances into the space between the antennomeres, consisting of either lubricants to facilitate antennal movements or antimicrobials to prevent infestation of this space since it cannot be easily cleaned by grooming. (2) Females might have deposited small amounts of this secretion during antennation of the brood cell walls (Strohm and Linsenmair, 1994/95) and larvae might have used it as an orientational cue to facilitate emergence from brood cells deep in the soil (thus making use of the orientational function of the secretion). Possibly some of the lipid containing secretion (Kaltenpoth et al., 2009) was taken up by the larva to facilitate cocoon spinning. (3) This would have selected for larger amounts of secretion and thus for an invagination of the cuticle to form a reservoir. (4) Now *Streptomycetes* from the surrounding soil might have invaded the glands, e.g., as commensals, and were applied to the brood cell along with the secretion that provides the orientational cue. These bacteria might have produced antibiotics to defend themselves against competing bacteria or fungi. Along with the lipids, larvae might have taken up some bacteria and incorporated them into their cocoons. (5) Due to the antibiotics, the bacteria might have protected the cocoons against mold fungi and, thus, enhanced the survival of the digger wasps’ offspring. This generated a positive feedback resulting in an increased size and complexity of the antennal glands and optimized the selection and cultivation of the most beneficial bacteria. (6) Consequently, the number of class III units to provide nutrients for the symbionts increased and they were arranged in acini. (7) To avoid leakage of the secretion an elastic plug formed to close the opening when the secretion was not needed. (8) To enable sequestration of nutrients for the bacteria the walls of the gland lumen differentiated. (9) Selection for even more secretion caused an additional increase of the gland lumen by a second lobe.

The scenario above implies that the orientational function has preceded the evolution of the symbiosis with bacteria. However, considering that *Streptomycetes* are ubiquitous in the soil and capable of utilizing different substrates for growth, including hydrocarbons and chitin (Radwan et al., 1998; Barabas et al., 2001; Schrempf, 2001), an alternative hypothesis might be conceived. The occurrence of *Streptomyces* spores in the brood cell may have led to colonization of the paralyzed honeybees that are being embalmed with large amounts of hydrocarbons as another antimicrobial strategy (Herzner et al., 2007; Herzner and Strohm, 2007). Some of these bacteria might have ended up on the cocoon and used the hydrocarbons as nutrients but also providing some additional protection from pathogenic fungi. This would have selected for mechanisms to accomplish transmission of the bacteria and to ensure that only suitable bacteria are secreted by evolving a compartmentalized space (Chomicki et al., 2020). That the bacteria ended up in the antennae might be due to the fact that beewolf females frequently antennate the surface of both, the brood cell and the bees. Thus, the *Streptomycetes* might have colonized the antennal cuticle, entered the space between the antennomeres, where their growth was then enhanced by secretions from novel or preexisting glands in this region. Subsequent steps like the formation of the reservoir, increased numbers of gland cells and so on would then follow as described above. This alternative scenario would imply that the orientational function of the antennal secretion evolved secondarily. For both hypotheses, the sequence of steps can be questioned and some steps might have evolved simultaneously.

Since the two hypotheses mainly differ with regard to the ancestral state from which the evolution of the symbiosis started, it would be worthwhile to investigate whether related species show any of the postulated first steps. A widespread occurrence of small antennal glands and their possible use for orientational signals would provide evidence for the first hypothesis. The occurrence of *Streptomycetes* in brood cells and on the cuticle in particular of the antennae and on the cocoons of related taxa would support the alternative hypothesis.

## Conclusion

Our results on the morphology of the antennal glands together with earlier studies and studies on the phylogeny of the bacterial symbionts of the Philanthini (Kaltenpoth et al., 2005, 2014a,b) suggest that these unique glands are a common feature in the tribe. Whether there are species or related genera that possess ancestral glands with less elaborate gland morphology or less sophisticated associations with *Streptomycetes* but have not yet established a symbiosis with the bacteria has to be analyzed by a more extensive analysis of the subfamily Philanthinae.

## Data Availability Statement

The original contributions presented in the study are included in the article/Supplementary Material, further inquiries can be directed to the corresponding author.

## Author Contributions

ES, WG, and MK conceived and designed the analysis, analyzed the data, prepared the figures, and wrote the manuscript. WG collected and processed the data. SM instructed and supervised data collection (μCT). All authors contributed to the article and approved the submitted version.

## Funding

This study was funded by the German Science Foundation (DFG, STR 532/2-1, STR 532/3-1) and the Volkswagenstiftung (VW I/82682).

## Conflict of Interest

The authors declare that the research was conducted in the absence of any commercial or financial relationships that could be construed as a potential conflict of interest.

## Publisher’s Note

All claims expressed in this article are solely those of the authors and do not necessarily represent those of their affiliated organizations, or those of the publisher, the editors and the reviewers. Any product that may be evaluated in this article, or claim that may be made by its manufacturer, is not guaranteed or endorsed by the publisher.
