# Supplementary material for: Comparative Morphology of the Symbiont Cultivation Glands in the Antennae of Female Digger Wasps of the Genus Philanthus (Hymenoptera: Crabronidae)
Source: Front Physiol. 2022 Jan 26;13:815494. doi: 10.3389/fphys.2022.815494 (PMC8826713; doi:10.3389/fphys.2022.815494)
Supplement: Supplementary file 1 [file Data_Sheet_1.PDF]

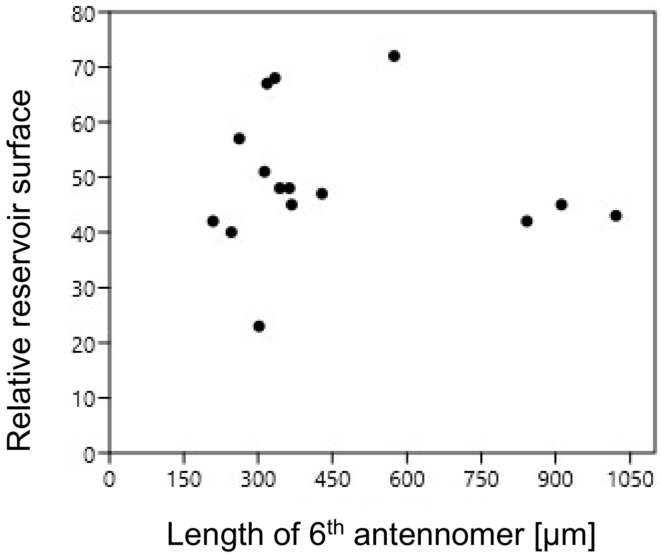

Supplementary Fig. 1 Relationship between the length of the antenna and the relative surface of the reservoir of the 6th antennomer for 15 *Philanthus* species. (Spearman correlation analysis: Rs = -0,08, N = 15, p = 0.78).

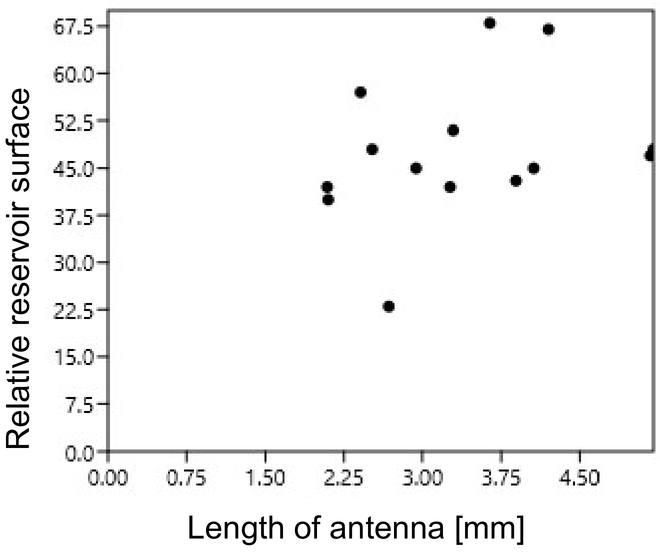

Supplementary Fig. 2 Relationship between the length of the antenna and the relative surface of the reservoir of the 6th antennomer for 15 *Philanthus* species. (Spearman correlation analysis: Rs = 0.28, N = 15, p = 0.32).

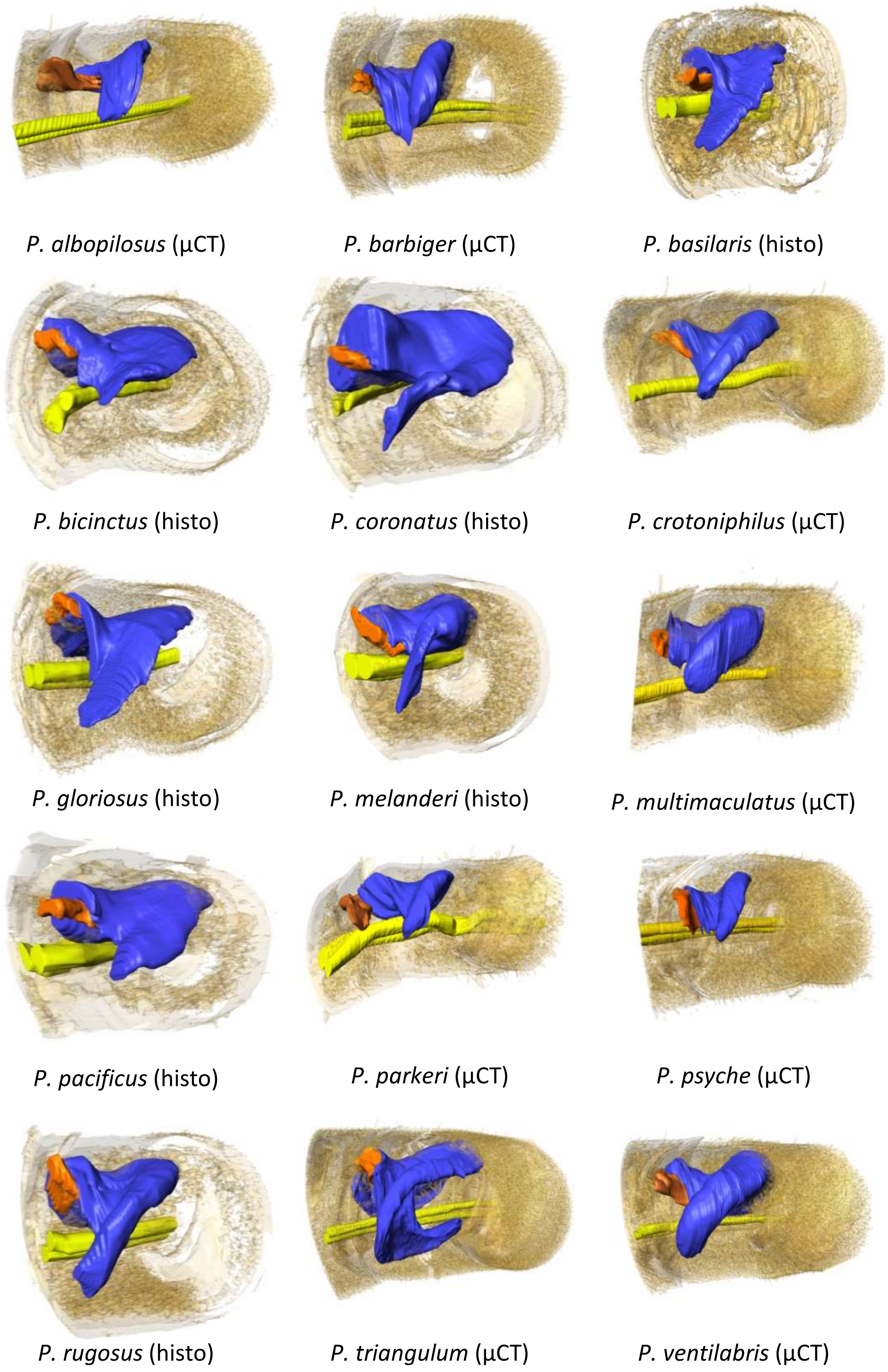

Supplementary Fig. 3: 3D-models of antennal glands in antennomere A6 of females of 15 *Philanthus* species. Reconstruction was either based on histological sections (histo) or on micro-computer tomography (µCT). Color code: yellow, antennal nerv; orange: flap; blue: gland reservoir; the outer antennomere cuticle is displayed half transparent.

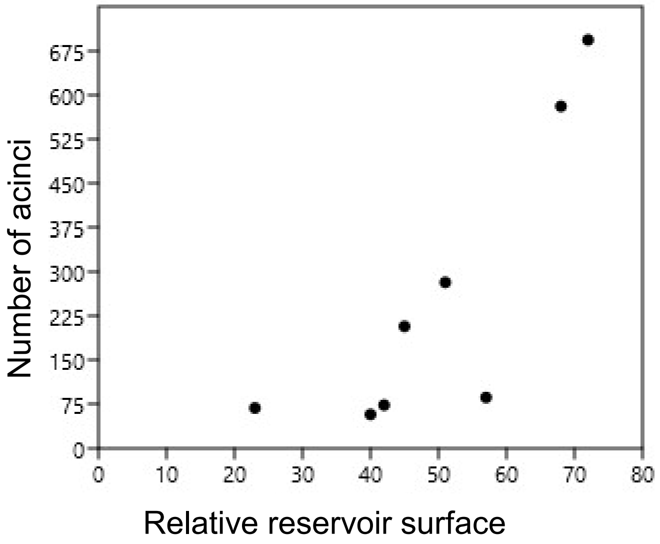

Supplementary Fig. 4 Relationship between number of acini and relative surface of the gland in the 6th antennomer for eight *Philanthus* species. The species that somewhat deviates from the trend is *P. multimaculatus* (lower right datapoint). (Spearman correlation analysis: Rs = 0.9, N = 8, p = 0.003).

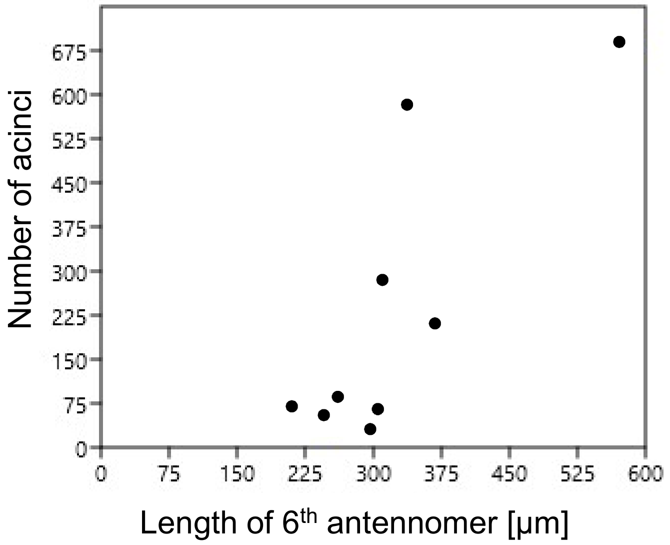

Supplementary Fig. 5 Relationship between the length of the 6th antennomer (µm) and the number of acini associated with the lumen of the 6th antennomer for eight *Philanthus* species. (Spearman correlation analysis: Rs = 0.81, N = 8, p = 0.018).
